# New and simplified method for drug combination studies by checkerboard assay

**DOI:** 10.1016/j.mex.2021.101543

**Published:** 2021-10-11

**Authors:** Pierangelo Bellio, Lorenza Fagnani, Lisaurora Nazzicone, Giuseppe Celenza

**Affiliations:** Department of Biotechnological and Applied Clinical Sciences, University of L'Aquila, L'Aquila, Italy

**Keywords:** Synergy, Antagonism, Antimicrobials, Minimal inhibitory concentration, MIC, EUCAST, CLSI, Drug-drug interaction

## Abstract

To evaluate the effect of two combined antimicrobial drugs, one method currently in use is the checkerboard assay in a 96-well microplate, which gives a good *in vivo* estimation of the drug-drug combination effect. Appropriate and consolidated methods are described in numerous scientific publications which are, however, in turn, laborious and time-spending, specifically for the setting of the 96-well microplate preparation. Each drug of every combination must be prepared and dispensed individually in several steps, often limiting its use in terms of consumed materials and working time. In our method, the strengths of the previous consolidated techniques are kept, although the toughness and the execution time are drastically reduced. No special laboratory apparatuses are needed. All the procedures of our method can be referred to the CLSI or EUCAST guideline. The method provides few main steps, which can be summarised in:

• Preparation of the microorganism inoculum and three concentrations of antimicrobial drugs.
• Easy dispensing of all reagents into the microplates with a multichannel pipette.
• Evaluation of the microorganism optical density (OD) by a microplate reader, and calculation of growth percentage for each of the 77 combinations.

## Graphical abstract

**Figure:**
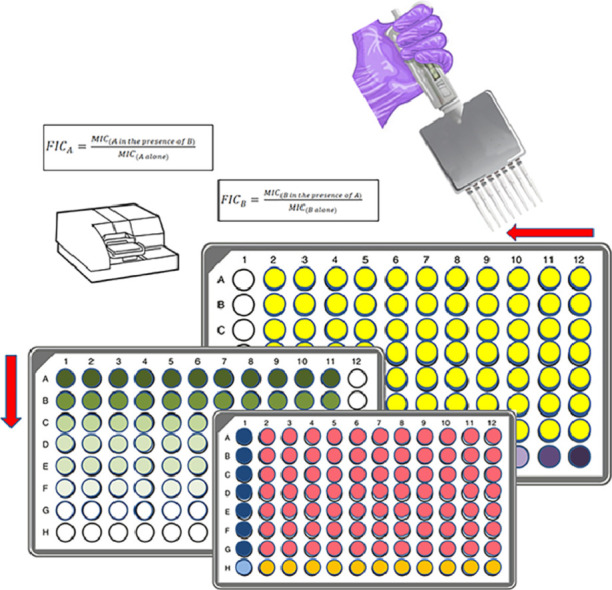

Specifications Table

**Table:** 

Subject Area:	Immunology and Microbiology
More specific subject area:	Microbiology
Method name:	**New and simplified method for drug combination studies by checkerboard assay**
Name and reference of original method:	L. Garcia (2014) Synergism Testing: Broth Microdilution Checkerboard and Broth Macrodilution Methods, in: Clinical Microbiology Procedures Handbook, 3rd Edition, American Society of Microbiology, 2014: pp. 140–162. 10.1128/9781555817435.ch5.12.
Resource availability	N.A.

## Introduction

When two or more drugs are tested together simultaneously, they can give different phenotypic effects: synergism, additivity, antagonism or indifference. To evaluate the effect of the combination between two antimicrobial drugs, the microbiologist uses the agar dilution method and the disk diffusion method. An additional method currently in use is the checkerboard assay in a 96-well microplate, which gives a good *in vivo* estimation of the drug-drug combination effect [1,2]. The latter uses a liquid medium and a two-dimensional, two-agents microdilution checkerboard to evaluate combinations of antimicrobial agents against organisms [1,2]. In the checkerboard assay, two antimicrobics are tested in double serial dilutions, and the concentration of each drug is tested both alone and in combination. Thus, it is possible to determine the effect of the individual drug, but above all, the effect produced by their combination. The nature of the interaction between the two antimicrobials is then determined either algebraically or geometrically [1]. The most used, appropriate and consolidated method is described in the Clinical Microbiology Procedures Handbook 3rd Edition [2], which is, in turn, laborious and time-spending, specifically for the setting of the 96-well microplate preparation. Each drug of every combination must be prepared and dispensed individually in several steps, often limiting its use in terms of consumed materials and working time. In our method, we use the strengths of the previously cited protocol but reducing its laboriousness and execution time, using only three concentrations of antimicrobial drugs, and dispensing the regents with a multichannel pipette, unlike a single channel pipette. Furthermore, no special laboratory equipment is needed.

All the procedures of our method refer to the CLSI [3] or EUCAST guideline [4] in some cases with some modifications. Though most drug interaction methods are not used in the clinical laboratory workflow, the numerous tests developed for this purpose are very useful in the practice of research laboratories.

Additionally, it is possible not only to test antimicrobial molecules with each other but also in combination with compounds that, normally, by themselves, do not have antimicrobial properties.

With the crisis generated in recent years, most antibiotics or antifungal drugs fail because of increasing antimicrobial resistance. The use of molecules in combination could be the key to the development of new formulations that do not necessarily require the discovery of new molecules.

## Method details

### Materials

• Isolated bacterial colonies.
• Two antimicrobics or compounds from stock solutions or powder.
• Mueller-Hinton broth (MHB).
• Cation-adjusted Mueller-Hinton broth (CAMHB).
• Double concentrated Cation-adjusted Mueller-Hinton broth (2XCAMHB).
• Diluents for the antimicrobial agent stock solutions.
• Sterile distilled reagent-grade water.
• Sterile 0.9% NaCl.
• V-shaped Sterile pipetting reservoir or sterile 90 mmor 100 mm diameter Petri dish.
• Electronic portable pipette controller or manual pipette controller.
• Sterile serological pipette: 2, 5, 10, 25, 50 mL.
• Manual or electronic single channel pipettes delivery tips (1000, 200 and 10 µL).
• Manual or electronic multichannel pipette (8 or 12 channel) deliver 200 µL tips.
• Sterile pipet tips: 1000, 200 and 10 µL.
• Sterile, clear round bottom, not treated and individually wrapped 96-well microplates with lid, or clear flat bottom, not treated and individually wrapped 96-well microplates with lid.
• 50, 15 and 1.5 mL sterile disposable plastic vials.
• 100 and 250 mL microbiological glass flask.
• Lids for glass flasks or bacteriological hydrophobic cotton.
• Glass beaker.

### Equipment

• Autoclave.
• Vortex mixer.
• Magnetic stirrer.
• 35±2°C ambient air orbital shaker-incubator.
• 35±2°C ambient air incubator.
• 96-well microplate reader equipped with a 600 nm optical filter or close to 600 nm.

## Procedure

**Day 1**

### Preparation of culture media, drug/compound stock solutions and microbial inoculum

#### Preparation of culture media

1. Weigh an appropriate amount of powdered culture medium.
2. Using a magnetic stirrer, dissolve the powder into deionised water until the required volume is reached (See recipe reported on the package insert or in the datasheet) and then sterilise by autoclaving at 121°C for 15 min.

The culture media required are:

• 10 mL of MHB.
• 50–100 mL of 2 × CAMHB.

#### Preparation of 100 mL of sterile saline solution (NaCl 0,9%)

1. Using a magnetic stirrer, dissolve 0.9 g of NaCl into 100 mL of deionised water and then sterilise by autoclaving at 121°C for 15 min.

#### Preparation of antimicrobial or compound stock from powder

1. Weigh an appropriate amount of powder of drug A and drug B and dissolve each one into its appropriate solvent as requested by CLSI in the chapter: “Solvents and Diluents for Preparing Stock Solutions of Antimicrobial Agents” [3] or as recommended by the EUCAST guidelines [4]. If the antibiotic or compound is not listed in the guidelines, use the solvent that keeps the compound more stable (This information is often available in the datasheet).

#### Preparation of microbial inoculum

1. Withdraw with a sterile loop an isolated colony from a nutrition agar plate.
2. Inoculated the colony into a 10 mL of MHB.
3. Grow the bacteria at 35±2°C in an orbital shaker incubator at 200 rpm under aerobic conditions for 18 h ±2.

*Note: After 18 h ±2 of incubation, under the condition indicated, the culture will reach the concentration of ∼ 10^9^ CFU/mL.*

**Day 2**

### Preparation of plate for the two-drugs interaction measurement by checkerboard assay

1. Place 100 μL of the 2 × CAMHB medium in a V-shaped reservoir or sterile Petri dish and dispense into each well of the microplate, by multichannel pipette, proceeding by column from left to right or vice versa.

### Preparation and dispensing of drug A

**Fig. 1:**
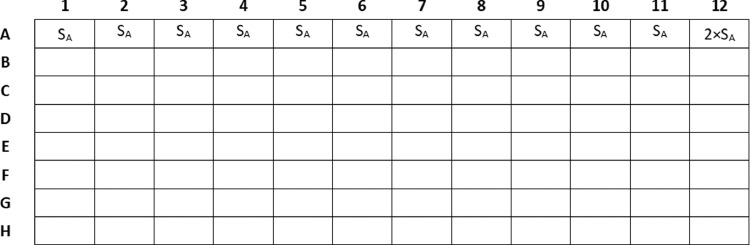
Disposition of drug A in the 96-well microplate.
4. Dispense 100 μL of 2 × S_A_ into well A12. (Fig. 1)

*Note: The purpose of adding the Stock solution 2 × S_A_ is to keep constant the concentration of drug A during the next serial dilution (point 9).*

1. Prepare 1.5 mL of stock solution (S_A_) in 2 × CAMHB medium to obtain a solution 4-fold more concentrated than the highest concentration of the drug A to be tested.
2. Prepare 500 μL of 2 × stock solution (2 × S_A_) in 2 × CAMHB medium to obtain a solution 8-fold more concentrated than the highest concentration of the drug A to be tested.
3. Dispense 100 μL of S_A_ into row A from columns 1–11. (Fig. 1)

### First serial dilution of drug A

1. Dilute the drug A from row A to G using a multichannel pipette set to 100 μL and discard the remaining volume from row G.

**Fig. 2:**
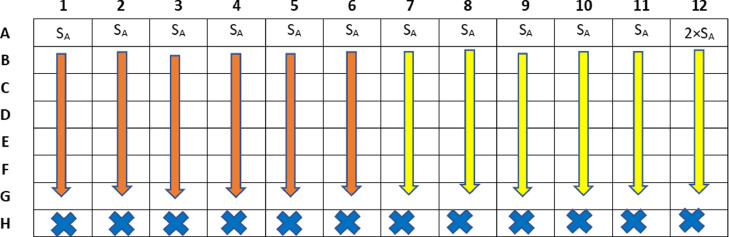
Dilution of drug A in the 96-well microplate by multichannel pipette.

*Note: With an 8-channel pipette, dilute in two steps using 6 tips. The first from the 1–6 and then from 7 to 12. When starting again with the second step, change the tips (* Fig. 2 *).*

### Preparation and dispensing of drug B

**Fig. 3:**
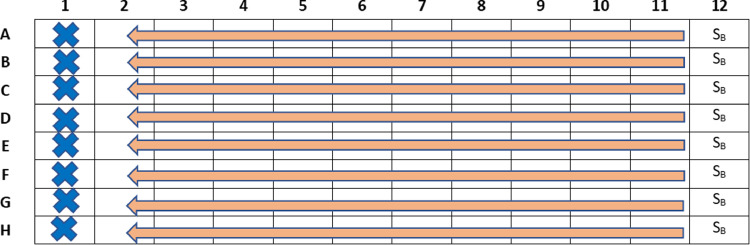
Disposition of drug B in the 96-well microplate and dilution by multichannel pipette.

1. Prepare 1.0 mL of stock solution (S_B_) in 2 × CAMHB medium to obtain a solution 4-fold more concentrated than the highest concentration of drug B to be tested.
2. Dispense 100 μL of S_B_ into each column 12 wells. (Fig. 3).

### Second serial dilution of drug B

1. Dilute the drug B Column 12–2 using a multichannel pipette set to 100 μL and discard the remaining volume taken from column 2. (Fig. 3).

### Plate Inoculation

1. Prepare from 10 to 40 mL of bacterial inoculum 10^6^ CFU/mL in 0,9% NaCl, from the culture prepared the day before (see chapter: Preparation of microbial inoculum).
2. Place 100 μL of inoculum in a V-shaped reservoir or sterile Petri dish and dispense into each well of the microplate using a multichannel pipette.

*Note:*

Before diluting the bacterial culture at step 10, mix by vortex for at least 20 s. The final inoculum concentration in the microplate will be 5 × 10^5^ CFU/mL.

A mirror plate, without bacteria and with the same reagents, should be implemented to obtain no-growth control. Moreover, the mirror plate is useful as O.D. background in the data analysis (i.e., when compound precipitates may appear during the incubation).

### Plate Incubation

1. Place the microplate with its cover into a static incubator at 35±2°C for 18±2 h.

*Note: If there should be an excessive variation in volume during incubation, it is possible to seal the microplate by sealing film. Also, a tray with water can be added to the incubator to prevent the dry environment favouring evaporation from the plate.*

**Day 3**

### Optical analysis by a microplate reader

1. Mix the contents of the wells using a multichannel pipette.
2. Place the microplate in a microplate reader.
3. Read the optical density (OD) at 600 nm or around 600 nm (e.g. 595 or 605).

### Data analysis

From the optical density, it is possible to calculate the percentage of growth for each well.

**Fig. 4:**
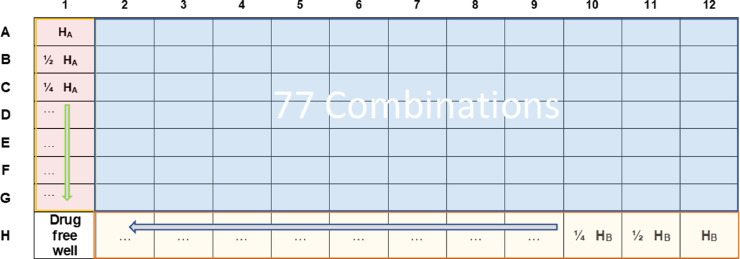
The final setting of 96-well microplate.

Based on the distribution of the reagents that have been made, the plate is organised as follows (Fig. 4):

■ In column 1 from A to G wells, there are the double serial dilutions of drug A. A1 is the well where the drug A has the highest concentration (H_A_).
■ In the raw H from columns 12–2, there are the double serial dilutions of drug B. H12 is the well where the drug B has the highest concentration (H_B_).
■ The well H1 represent the bacterial growth control without drugs (100% growth).
■ All the other wells of the microplate represent all the 77 possible combinations formed by the combination of the serial dilutions of each antibiotic.

The percentage of growth in each well was calculated as:

$$\frac{OD_{drug\;combination\;well}-OD_{background}}{OD_{drug\;free\;well}-OD_{background}}\times \;100$$

*Note: OD_background_ is the value of OD where no growth is appreciable or a value previously determined using the same method, where the reagents without bacteria were placed*.

*From the data, it is also possible to calculate the Minimal Inhibitory Concentration (MIC) for each combination. MIC is defined as the lowest drug concentration, which reduces bacterial growth by more than 80%.*

### Drug interaction models

Several models can be used to analyse the data obtained from the new method of checkerboard assay and assess the nature of *in vitro* interaction.

Some applied examples can be extrapolated from the following publication:

[5] Segatore, B. et al*.* (2012) Phytomedicine, 19 (3-4), pp. 341-347.
[6] Celenza, G. et al. (2012) Phytomedicine, 19 (7), pp. 596-602.
[7] Bellio, P. et al. (2015) Phytomedicine, 22 (2), pp. 223-230.

In these studies, data were analysed by nonparametric models based on the Loewe additivity model and the Bliss Independence theory [8].

## Declaration of Competing Interest

The authors declare that they have no known competing financial interests or personal relationships that could have appeared to influence the work reported in this paper.
